# Amplifying the dielectric constant of shellac by incorporating natural clays for organic field effect transistors (OFETs)

**DOI:** 10.55730/1300-0527.3603

**Published:** 2023-10-11

**Authors:** Sunwoo KIM, Çiğdem YUMUŞAK, Cristian Vlad IRIMIA, Mateusz BEDNORZ, Esma YENEL, Mahmut KUŞ, Niyazi Serdar SARIÇİFTÇİ, Bong Sup SHIM, Mihai IRIMIA-VLADU

**Affiliations:** 1 Department of Chemical Engineering, Inha University, South Korea; 2 Program in Biomedical Science & Engineering, Inha University, South Korea; 3 Linz Institute for Organic Solar Cells (LIOS), Institute of Physical Chemistry, Johannes Kepler University Linz, Linz, Austria; 4 Department of Chemical Engineering, Konya Technical University, Konya, Turkiye

**Keywords:** Shellac, natural clays, shellac-clays composite, natural dielectric

## Abstract

We demonstrate in this work the practical use of uniform mixtures of a bioresin shellac and four natural clays, i.e. montmorillonite, sepiolite, halloysite and vermiculate as dielectrics in organic field effect transistors (OFETs). We present a thorough characterization of their processability and film forming characteristic, surface characterization, elaborate dielectric investigation and the fabrication of field effect transistors with two classic organic semiconductors, i.e. pentacene and fullerene C60. We show that low operating voltage of approximately 4 V is possible for all the OFETs using several combinations of clays and shellac. The capacitance measurements show an improvement of the dielectric constant of shellac by a factor of 2, to values in excess of 7 in the uniform mixtures of sepiolite and montmorillonite with this bioresin.

## 1. Introduction

The industrial and commercial potential of organic electronics was demonstrated in recent years by the massive advancement in OLED technology [1,2,3] and the recent surge of organic photovoltaics (OPVs), with reports of efficiencies approaching 20% for the classic organic photovoltaics in bulk heterojunction design, and even exceeding this value for perovskites. These landmarks make nowadays such developments suitable for both small scale and large-scale applications [4,5]. Nevertheless, despite the recently demonstrated impressive progress of electronics and sensors, the fabrication of next generation OLEDs, solar cells, and printed circuits (based on organic field effect transistors, OFETs), faces already challenges in terms of identifying novel higher-performance semiconductors, substrate and packaging materials, dielectrics, and processing conditions [6–11] to name just a few. Stable operation in air in the RF-regime of organic materials (i.e. megahertz and even higher frequencies) would support the development of many novel technologies capable to compete with silicon-based CMOS circuitry [8,12–18]. When such novel electronic constituents are combined with biosensing elements it will open the possibility for developing disposable diagnostic and drug-delivery technologies [19–29].

**Figure 1:**
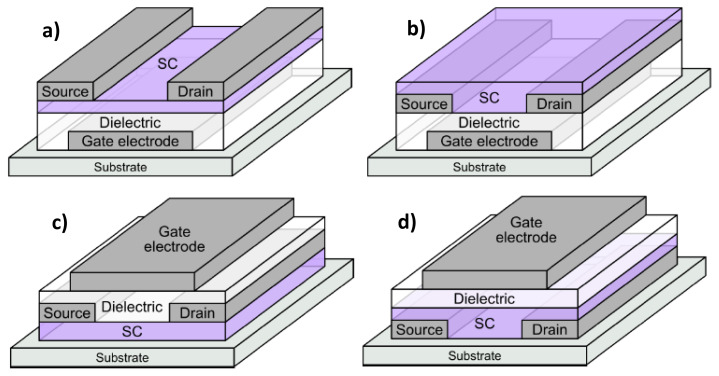
The four geometries of staggered (vertical) organic field effect transistors: a) bottom gate-top contact, BGTC; b) bottom gate-bottom contact, BGBC; c) top gate-top contact, TGTC; d) top gate-bottom contact, TGBC.

The rapid growth of electronic and sensors devices applications led to an increased demand for green and biodegradable materials and sustainable solutions for waste management to reduce the environmental impact of E-waste [30,31]. Organic field-effect transistors (OFETs) are developed as a promising electronic device for various applications on flexible, conformable, even imperceptible substrates [32,33]. OFETs consist of an organic semiconducting layer sandwiched between three electrodes (gate, source and drain), with an insulating layer acting as a dielectric between the gate electrode and the semiconductor. They usually are produced in one of the 4 possible staggered (vertical) geometries, shown schematically in Figure 1.

**Figure 2:**
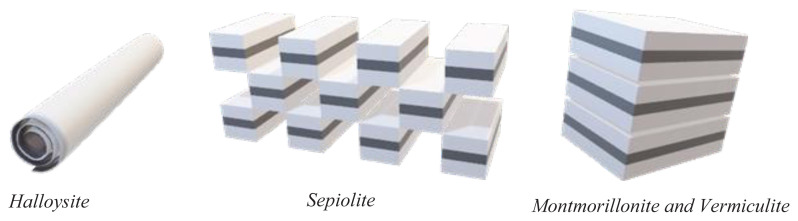
Schematic of the structural arrangement of clays.

While traditional FETs use inorganic materials like silicon dioxide (SiO_2_) [34], aluminum oxide Al_2_O_3_ [35,36] and other metal oxides [37], or other metal oxides for the and insulating layer, and silicon (Si) or other metal oxides like ZnO [38] for example for the semiconducting layer, there has been growing interest from the scientific community in developing OFETs with eco-friendly materials that are renewable, biodegradable, and nontoxic [39,40,41]. Clay minerals have shown great promise as a polymer filler due to their abundance and easy availability, however their implementation in electronic devices is yet to be accomplished [42,43]. In recent years nevertheless, various types of clay minerals, such as montmorillonite (MMT) [44,45], sepiolite [46,47], halloysite [48,49], and vermiculite [50], have been investigated as polymer composites in various perovskite solar cells and capacitors formulations. The properties of clay minerals vary depending on their chemical composition and structure, which can be influenced by their source or postprocessing [51]. MMT, sepiolite, halloysite, and vermiculite investigated here, are all types of clay minerals with distinct characteristics and structures, depicted schematically in Figure 2. Halloysite is a tubular clay mineral, consisting of a layer of alumina and silica sheets rolled into a cylinder, with a hollow interior [52], as shown schematically in Figure 2a). It is a type of clay mineral that belongs to the kaolin group, named after the French geologist Omalius d’Halloy, who first described the mineral in 1826. Halloysite is formed by the weathering and alteration of volcanic ash deposits and is commonly found in association with other clay minerals such as kaolinite and montmorillonite. It has mesopores with typical dimension of approximately 2–50 nm and macropores with a typical dimension in excess of 50 nm. Naturally formed halloysites exist mostly as tubular, but spherical and planar structures are possible also in nature [53,54]. The chemical formula is described to be Al_2_Si_2_O_5_(OH)_4_ 2H_2_O and generally observed in octahedral structure [55]. Halloysite has a relatively high specific surface area more than kaoline but less than montmorillonite.

Sepiolite, on the other hand, is a fibrous clay mineral with a three-dimensional structure, consisting of silicate chains linked by magnesium and aluminum ions, with a chemical formula Mg_4_Si_6_O_15_(OH)_2_·6H_2_O [56]. Sepiolite is a hydrous magnesium silicate mineral that belongs to the clay mineral group. It is also known as “meerschaum” in German or “sea foam” in English, originally named by Abraham Gottlob Werner in 1788. The name sepiolite originates from Ernst Friedrich Glocker in 1847. It is a white or grayish, soft mineral with a fibrous texture that makes it ideal for use as an absorbent material. It is also highly porous, with a large internal surface area due to its mesoporous fiber like structure, which makes it an excellent filter media for water purification [57]. The length dimensions of the microtubular structure of sepiolite are about 1μm to 2 μm, a width of 10 nm and contain open channels with dimensions of 3.6 Å × 10.6 Å running along the axis of the particle, resulting in a high pore volume and extremely high adsorption capacity. Sepiolite has a density varying from 0.988 to 1.279 g/cm^3^ [58,59].

MMT is a layered silicate mineral with a two-dimensional structure, consisting of an octahedral alumina sheet sandwiched between two tetrahedral silica sheets; basically, it is considered a dioctahedral 2:1 phyllosilicate [60]. The MMT clay has a plate-shaped appearance with an average diameter around 1 μm and a typical thickness of 0.96 nm. A characteristic of this clay is swelling when incorporating water in between the loosely bound individual crystal planes; hence montmorillonite is a typical component of swelling soil. The chemical formula of MMT is described as (Na,Ca)_0.33_(Al,Mg)_2_(Si_4_O_10_)(OH)_2_·nH_2_O.

Vermiculite has also a layered structure, similar to MMT, but with a higher degree of structural disorder and a greater degree of expansion when heated, due to the presence of interlayer water molecules [61]. Vermiculite is a naturally occurring mineral that belongs to the phyllosilicate group of minerals, with a chemical formula (Mg,Fe^2+^,Fe^3+^)_3_[(Al,Si)_4_O_10_] and an intercalated repeated unit (OH)_2_·4H_2_O. It is formed from the alteration of certain types of mica and its expansion when heated recommended vermiculite for many practical applications. The expanded form of vermiculite is lightweight, fire-resistant, absorbent, and has excellent insulation properties, which make it a popular material in many industries [62]. Vermiculite is commonly used in construction as an insulating material for walls, roofs, and floors. It is also used in horticulture as a soil amendment, as it improves water retention and aeration in soil. Vermiculite is also used in the manufacturing of fireproof boards, brake pads, and high-temperature insulation.

Natural resin shellac is a secretion produced by the lac bug *Kerria Lacca* in Southeast Asia. This resinous material is composed of polyhydroxy acids and waxes and is harvested and processed for a variety of applications [63,64]. The main producer of shellac in the world are India, Thailand and China, and shellac is in fact the only industrially relevant bioresin in the world, with applications in food industry as citrus food preservation, chocolate and candies glazing or in pharmaceutical field as taste masking of bitter pills [65,66,67]. Shellac is found in nature in many colors, ranging from a very light yellow to a very dark brown, with many varieties of yellow, orange, brown and red in between. The color of shellac is influenced by the sap of the host tree on which the lac bug lives and feeds, the time of harvest in the calendar year, and it also varies depending also on the amount of rainfall experienced by the host tree in the respective season. Shellac was also used for centuries as a versatile coating material due to its unique properties, including excellent adhesion, water resistance, and UV stability. It is commonly used as a varnish or protective coating for flooring, furniture, and musical instruments. In addition, shellac, as an eco-friendly electronic material, was demonstrated by our group in OFETs as a substrate and dielectric layer, and thus, is an electronic material [68].

In this study, we demonstrate clay-shellac composites as a dielectric layer with high dielectric constant in OFETs. We combined shellac as the dielectric in OFETs with four types of nanoclay minerals as the filler materials. We have used ammonia as a dispersant to improve the compatibility between shellac and clay minerals. Pentacene and C_60_ were used as p-type and n-type semiconductors, respectively. We investigated the effect of the type of clay mineral on the performance of OFETs.

## 2. Materials and methods

**Figure 3:**
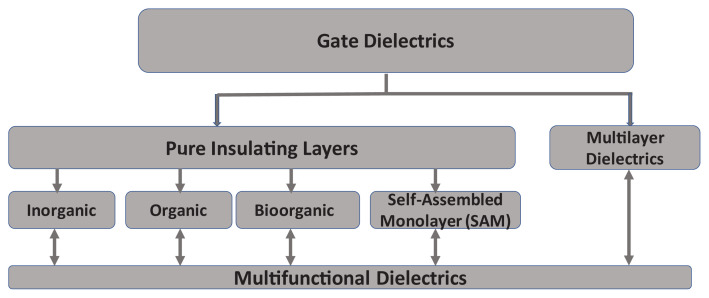
Typical dielectric classes employed in organic electronic devices.

Out of the four possible staggered OFET geometries exemplified in Figure 1, we employed in this work the BGTC structure, that is in fact the typical structure successfully employed in laboratory research that does not give rise to contact resistance [69]. From the possible insulating layers employed until now in organic electronics (see Figure 3), a novel structure of Bioorganic + İnorganic particles, namely bio-resin shellac and natural, inorganic clays, in a similar way to our previous demonstration of pinaceae resins and inorganic nanoparticles of TiO_2_ [70].

**Figure 4:**
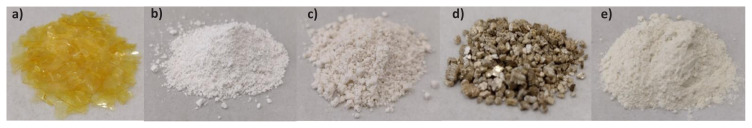
Photography of the employed materials in this study: a) shellac, b) halloysite; c) sepiolite; d) vermiculite; e) montmorillonite (MMT).

The schematic of the four clays materials is presented in Figure 2, whereas a photograph of the materials employed in this study is presented in Figure 4. Shellac material for this study was offered to us by AF Suter & Co. Ltd. It is the commercial grade HS700K.

### 2.1. Preparation of clay/shellac solutions

Each clay was added to water at a concentration of 1 mg/mL, and then dispersed using ultrasonication for 24 h. After allowing about 2–3 h for the larger conglomerates to deposit at the bottom of the flask, a well-dispersed solution was collected from the upper part of the mixture. Shellac on the other hand was added to ethanol at a concentration of 25 mg/mL, and then dissolved completely by stirring at 50°C for 2 h. The dissolved shellac solution and well-dispersed clay solution are mixed in a 1:1 ratio, by extracting 2 mL of each, and adding 50 μL of a 30% ammonia solution to allow shellac to be finely dissolved in the water component of the solution. The prepared mixtures were ultrasonicated for another 24 h before further use.

### 2.2. Devices fabrication

Glass substrates were cleaned by sonicating them in a 0.2% Hellmanex cleaning agent in water solution for 30 min, followed by 30-min sonication in distilled water. Aluminum was deposited as a bottom gate electrode onto the substrate via physical vapor deposition to a thickness of 80 nm. Subsequently, the prepared shellac-clay solution was deposited in a thin dielectric layer by spin-coating portions of 100 μL mixtures onto each individual glass substrates at speeds of 800 rpm for 10 s, and then at 1650 rpm for 20 s. Since the article deals with the report of novel dielectric materials, we counterbalanced it by selecting to work with two well established semiconductors, i.e. n-type fullerene, C_60_ and p-type pentacene. Both organic semiconductors (C_60_ and pentacene) were deposited using the thermal evaporation method on shellac-clay layer, in a custom-made organic evaporation system (Vaksis R&D and Engineering, Ankara, Türkiye). For the source and drain electrodes, aluminum, and gold, each of a thickness of 80 nm were deposited through physical vapor deposition in a glove box.

**Figure 5:**
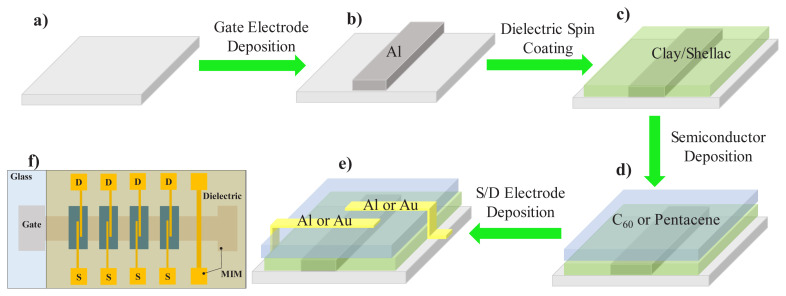
a–e) Schematic of the fabrication steps for the organic field effect transistors; f) schematic of a fully fabricated glass slide containing four field-effect transistors. The source and drain contacts were aluminum for the fullerene (C_60_) and gold for the pentacene semiconductor, respectively.

The mask design used in this study allowed to pattern 4 individual areas of organic semiconductors as shown schematically in Figure 5. The source and drain mask produced 4 sets of contacts for the 4 semiconductor patches and one continuous electrode that was used for the measurement of the dielectric capacitance between itself and the underneath gate electrode. The respective capacitance was the one used individually for each slide for the calculation of the field effect mobility of the 4 OFET devices on that slide. The source and drain dimensions of the channel used in this study were 2 mm in width (W) and 25 μm in length (L). The gate electrode width of 2 mm represents the width of the semiconductor channel, as the schematic in Figure 5 shows. For the calculation of each of the two semiconductors field effect mobility, the specific capacitance of the dielectric (expressed in nF/cm^2^) was employed in the mobility formula [71,72], by dividing the capacitance measured on the MIM structure of the OFETs slide (see Figure 5) to the area of the two overlapping electrodes, i.e. 0.8 mm^2^.

The dielectric function and the dielectric capacitance were measured in a follow up study on a Novocontrol Broadband Impedance Analyzer (Novocontrol GmbH), by measuring on the MIM structure of the transistor slide (see Figure 5). For each individual clay and shellac combination and each organic semiconductor, we casted thin films via spin coating procedure described above and fabricated 2 batches of 6 slides per each batch, amounting to a total 48 OFET devices and 12 MIM samples (see the mask design in Figure 5) for obtaining a good statistic of the measurements.

## 3. Results and discussion

Our attempt to fabricate films of clays cast from water dispersions and employ them as dielectric layer proved unsuccessful, mainly because of the extreme porosity and the inherently coarse nature of the respective materials. We then focused our attention on creating films from the mixtures of the respective clays and the animal resin shellac that is a material well known for its easy processability and smooth film formation [68]. We pursued two approaches in this regard: (1) making homogeneous mixtures of clays and shellac and use such mixtures for casting the dielectric layer via spin-coating, and (2) depositing the two materials (clay and shellac) sequentially, basically generating a bilayer structure. Although working decent for MMT-shellac and vermiculite-shellac bilayers, the second method did not produce reproducible results for the other two clays in bilayer structures with shellac; therefore, we decided to focus our attention on working exclusively with homogeneous mixtures of clays and shellac of the formulation described in the experimental section.

**Figure 6:**
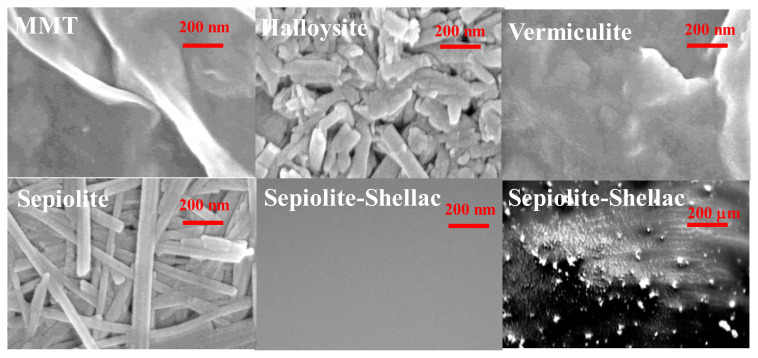
Scanning electron microscopy investigations of the natural clays in water. Top line, from left to right: films of MMT, halloysite and vermiculite cast from water dispersion; bottom line, left panel: film of sepiolite cast from water dispersion; middle panel: film of the uniform mixture of sepiolite and shellac seen at 200 nm magnification: right panel: the same film seen at 200 μm magnification.

Scanning electron microscopy (SEM) of cast films of clays from water solutions on conducting indium tin oxide electrode is presented in Figure 6 at a magnification of 200 nm, with the respective clays labeled on each SEM image. As shown by the respective scans, all the clays dispersed in water, either need a filler material in order to seal their voids, or themselves need to be used as fillers in a more robust, good film forming substance like shellac. We show as a support of this argument, in Figure 6 bottom row, the example of a composite film of sepiolite and shellac. The film cast from respective mixture has featureless surface at 200 nm and even at 2 μm magnification (data not presented, to avoid repeating the featureless image at 200 nm magnification). However, at a very large magnification of 200 μm it is visible that lumps of coalesced sepiolite are still present in the mixture. Nevertheless, as it will be seen in the transistor characterization section, these lumps of clays do not affect the OFET performance given the width of the OFET channel of 2 mm and its length of 25 μm.

**Figure 7:**
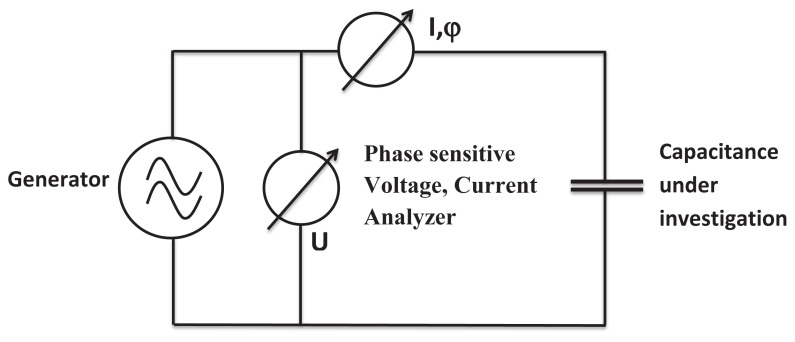
Schematic of the circuit employed in the impedance measurement.

**Figure 8:**
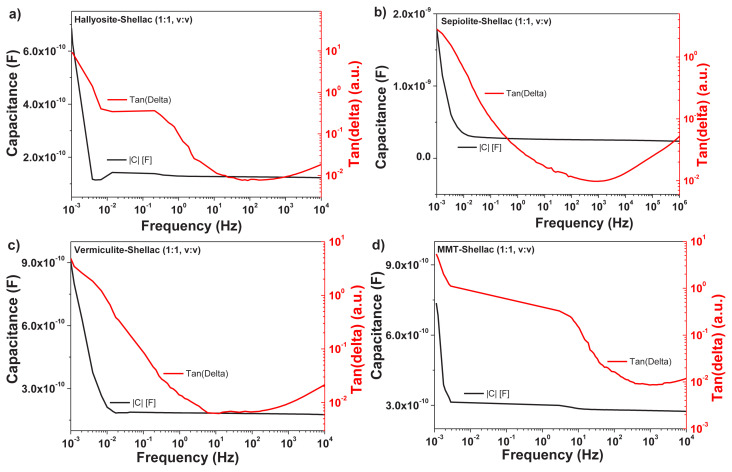
Impedance spectroscopy for the clays-shellac homogeneous mixtures in the frequency range of 10 kHz to 1 mHz; a) halloysite-shellac; b) sepiolite-shellac; c) vermiculite-shellac; d) MMT-shellac. The contact area of the MIM electrodes was 0.8 mm2: 2 mm is the width of the bottom electrode, i.e. the width of the OFET gate, and 0.4 mm is the width of the top floating electrode, as shown in the schematic of Figure 5f). The film thicknesses are listed in Table 1.

Impedance spectroscopy is a valuable tool employed to investigate processes that occur at interfaces, particularly the variations in physical properties of the system (i.e. electrical, compositional, crystallographic, or even mechanical). With respect to electrical changes in the system under investigation, impedance spectroscopy studies the polarization effect on the electrical conductivity of the system over a wide range of frequencies [73,74]. We performed impedance spectroscopy investigations of homogenous films of clays and shellac in 1:1, v:v ratio, cast between two aluminum electrodes is performed with a circuit design exemplified in Figure 7. The impedance scan of the four clays and shellac in the frequency window spanning between 1 MHz and 1 mHz is displayed in Figure 8. The values of the film thicknesses and the measured capacitances (at 1000 Hz) and dielectric constant are presented in Table 1. Given the variation in the films thickness within each batch of investigated clays-shellac, and the corresponding measured capacitance of each MIM structure, the dielectric constant also produced a range of values, ranging with similar dispersity for all the 4 mixtures of clays and shellac. For the sepiolite and MMT mixtures with shellac, a dielectric constant as high as 7.5 and 7.7 respectively was measured for several MIM devices, whereas vermiculite and halloysite produced dielectric constant films of 5.0 and 5.3 respectively when mixed in 1:1 (v:v) ratio with shellac. The entire set of calculations is presented in Table 1. What is important to note is that the addition of clays fillers into shellac does not change the overall behavior of the bioresin. Indeed, the clays-shellac films show a uniform capacitance down to the entire low frequency spectrum investigated. The change in capacitance of the respective films from kilohertz to millihertz is merely a factor of 3 increase in the millihertz frequency range compared to the kilohertz counterpart, which is common to any of the outstanding organic dielectrics reported elsewhere [75,76,77]. In the same time the loss angle measurement (depicted as a red type line in each graph in Figure 8) does not show any relaxation (dome shape) in the low frequency range, proving as in the case of shellac alone [68] that shellac filled with clays microparticles does not show any type of ionic behavior.

**Figure 9:**
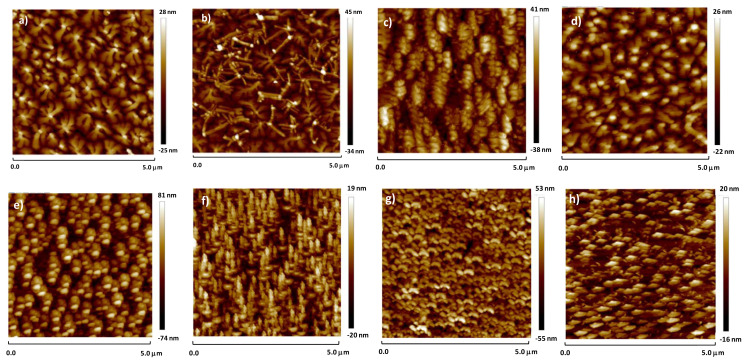
Atomic force microscopy images of the organic semiconductors grown on the clays-shellac dielectric layers; a) pentacene on halloysite-shellac; b) pentacene on sepiolite-shellac; c) pentacene on vermiculite-shellac; d) pentacene on MMT-shellac; e) C_60_ on halloysite-shellac; f) C_60_ on sepiolite-shellac; g) C_60_ on vermiculite-shellac; h) C_60_ on MMT-shellac.

Atomic force microscopy images of the organic semiconductors pentacene and C_60_ are analyzed by atomic force microscopy (AFM) using tapping mode Figure 9. The AFM topographic images for both semiconductors show uniform grains grown, however these grains are significantly different for each combination of clay-shellac dielectric and organic semiconductor. The grains of pentacene show dendritic type of growth on all 4 clays-shellac dielectric layers and have uniform sizes of about 800 nm, whereas the C_60_ grains are much smaller and elongated, with dimensions in the range of 100 nm.

**Figure 10:**
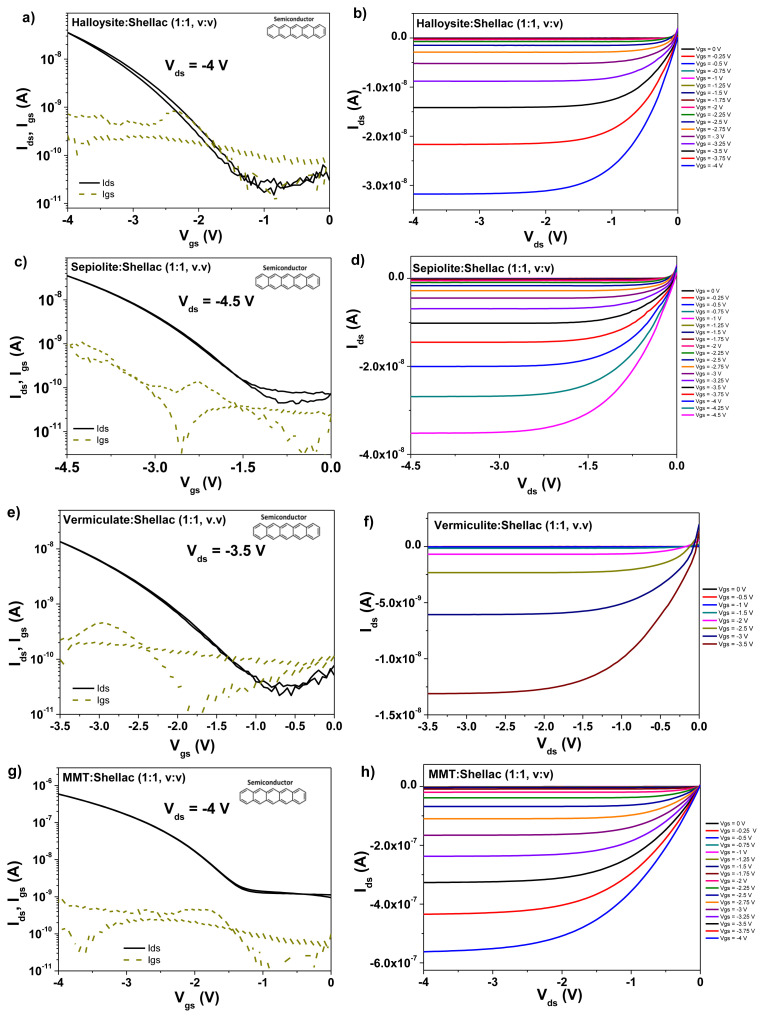
Transfer and output characteristics of OFET devices with clays-shellac homogeneous mixture as gate dielectrics and pentacene semiconductor. a–b) Halloysite-shellac; c–d) sepiolite-shellac; e–f) vermiculate-shellac; g–h) MMT-shellac.

**Figure 11:**
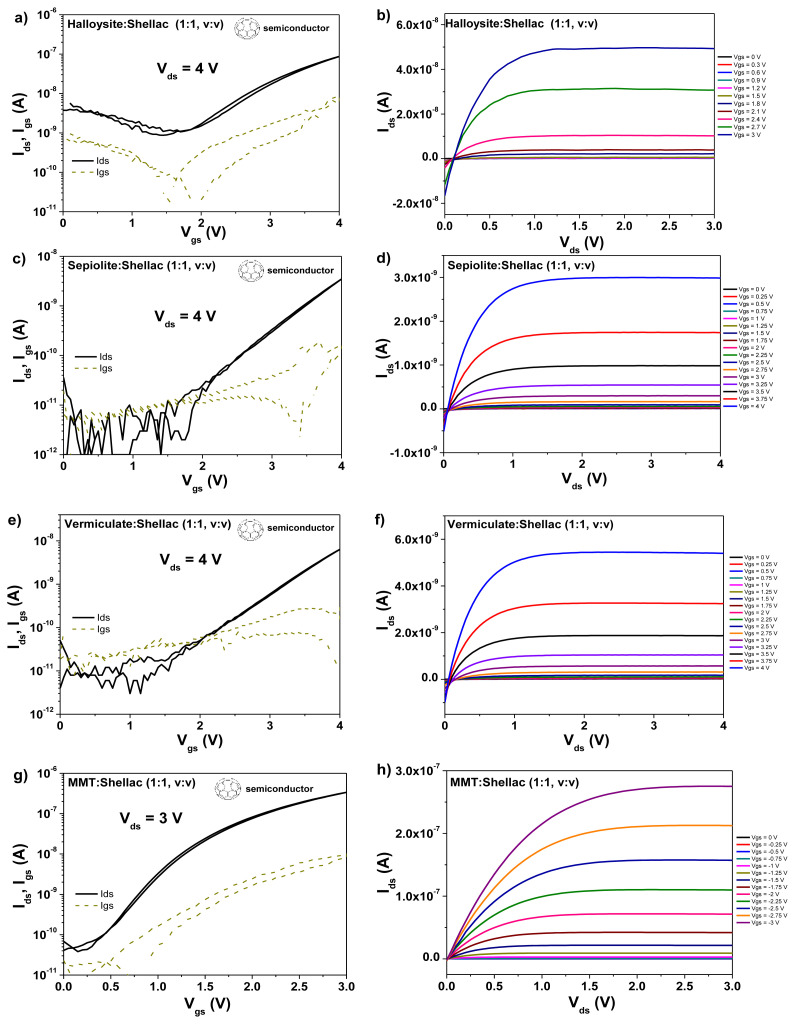
Transfer and output characteristics of OFET devices with clays-shellac homogeneous mixture as gate dielectrics and C_60_ semiconductor. a–b) Halloysite-shellac; c–d) sepiolite-shellac; e–f) vermiculate-shellac; g–h) MMT-shellac.

OFETs with homogeneous mixtures of clays and shellac as gate dielectric layers were fabricated according to the procedure detailed in the experimental section, in a staggered, bottom-gate top contact geometry (BGTC). We used pentacene and C_60_ as organic semiconductors and capped the semiconductors with gold in the case of pentacene and in aluminum in the case of C_60_, as source and drain electrodes. The results of the transistors measurements with pentacene semiconductor are presented in Figures 10a–10h, whereas the corresponding results with C_60_ as organic semiconductor are presented in Figures 11a–11h. In both cases the devices with clays-shellac dielectrics worked at operating voltages not higher than 4.5 V, and displayed hysteresis-free behavior. In addition, the devices showed good saturation of the source-drain current, visible especially in the output characteristics. Given the preferential growth of pentacene in much larger grain sizes than C_60_ on the dielectric films of clays-shellac, it is not surprising that the overall performance of the pentacene based transistors is better compared to the fullerene counterparts, as Table 2 shows, especially when considering the field effect mobility of the two organic semiconductors. If one particular winner of the 4 clays has to be highlighted, then according to the data displayed in Figures 10 and 11 as well as Table 2, this seems to be montmorillonite (MMT). Indeed, the respective clay and shellac showed the best performance overall, in terms of semiconductors mobility, threshold voltage and subthreshold swing.

## 5. Conclusion

We have demonstrated in this study a possible avenue to make use of natural clays for the fabrication of organic electronic devices (i.e. OFETs). Given their inherently porous feature, we selected natural resin shellac as the matrix for the clays and fabricated field effect transistors with both a classic p-type semiconductor, pentacene and a classic n-type semiconductor, C_60_. The results of the dielectric measurements show that the 1:1 mixture per volume of clays and shellac are a very good and robust dielectric choice for the fabrication of organic field effect transistors. Moreover, the dielectric constant of the shellac can be significantly increased (doubled) from a typical value of approximately 3 by the addition of clay fillers [68,78]. With more attention needed to improve their processability, this work demonstrates a new class of fully natural dielectric compounds, i.e. natural clays, that have inherently a high dielectric constant. Such novel materials open the door for the fabrication of sustainable, cost-conscious electronic devices.

## Figures and Tables

**Table 1:** Dielectric properties of the investigated clays:shellac composite films.

Investigated dielectrics	Thickness (nm)		Dielectric constant	
	Min	Max	Min	Max
MMT-shellac	140	203	5.3	7.7
Sepiolite-shellac	181	213	6.3	7.5
Halloysite-shellac	161	180	4.8	5.3
Vermiculite-shellac	146	185	3.9	5.0

**Table 2:** Transistors parameters for the OFETs with clays-shellac dielectric and pentacene or C_60_ as organic semiconductors.

OFETs	Specific capacitance (nF/cm^2^)	Field effect mobility (cm^2^/Vs)		Threshold voltage (V)	Subthreshold swing (mV/dec.)
		Hole channel	Electron channel		
Halloysite-shellac-pentacene	15.62	3.2 × 10^−^^2^	-	−2.1	698
Halloysite-shellac-C_60_	15.62	-	1.5 × 10^−^^2^	2.1	113
Sepiolite-shellac-pentacene	19.75	1.0 × 10^−^^2^	-	−2.0	920
Sepiolite-shellac-C_60_	19.75	-	3.5 × 10^−^^3^	2.75	1270
Vermiculite-shellac-pentacene	22.37	6.2 × 10^−^^3^	-	−1.65	729
Vermiculite-shellac-C_60_	22.37	-	5.2 × 10^−^^3^	2.5	860
MMT-shellac-pentacene	33.5	0.1	-	−1.75	483
MMT-shellac-C_60_	33.5	-	8.6 × 10^−^^2^	0.85	365
